# Comparison of hypotension incidence between remimazolam and propofol in patients with hypertension undergoing neurosurgery: prospective, randomized, single-blind trial

**DOI:** 10.1186/s12871-024-02578-7

**Published:** 2024-06-04

**Authors:** Seung Ho Choi, Kyeong Tae Min, Eun Kyung Park, Sujung Park

**Affiliations:** 1 Department of Anesthesiology and Pain Medicine, Severance Hospital, Anesthesia and Pain Research Institute, Yonsei University College of Medicine, 50-1 Yonsei-Ro, Seodaemun-Gu, Seoul, 03722 Republic of Korea; 2 Department of Pediatric Neurosurgery, Severance Children’s Hospital, Yonsei University College of Medicine, Seoul, Republic of Korea; 3 Department of Anesthesiology and Pain Medicine, Asan Medical Center, University of Ulsan College of Medicine, Seoul, Republic of Korea

**Keywords:** Anesthetic induction, Remimazolam, Propofol, Hypertension, Drug therapy

## Abstract

**Background:**

Remimazolam, a newer benzodiazepine that targets the GABA_A_ receptor, is thought to allow more stable blood pressure management during anesthesia induction. In contrast, propofol is associated with vasodilatory effects and an increased risk of hypotension, particularly in patients with comorbidities. This study aimed to identify medications that can maintain stable vital signs throughout the induction phase.

**Methods:**

We conducted a single-center, two-group, randomized controlled trial to investigate and compare the incidence of hypotension between remimazolam- and propofol-based total intravenous anesthesia (TIVA). We selected patients aged between 19 and 75 years scheduled for neurosurgery under general anesthesia, who were classified as American Society of Anesthesiologists Physical Status I–III and had a history of hypertension.

**Results:**

We included 94 patients in the final analysis. The incidence of hypotension was higher in the propofol group (91.3%) than in the remimazolam group (85.4%; *P* = 0.057). There was no significant difference in the incidence of hypotension among the various antihypertensive medications despite the majority of patients being on multiple medications. In comparison with the propofol group, the remimazolam group demonstrated a higher heart rate immediately after intubation.

**Conclusions:**

Our study indicated that the hypotension incidence of remimazolam-based TIVA was comparable to that of propofol-based TIVA throughout the induction phase of EEG-guided anesthesia. Both remimazolam and propofol may be equally suitable for general anesthesia in patients undergoing neurosurgery.

**Trial registration:**

Clinicaltrials.gov (NCT05164146).

## Background

Hypertension is the most common concomitant disease encountered by anesthesiologists [1, 2]. In patients with hypertension, a rapid decline in blood pressure may occur during the induction phase [3, 4]. These patients are at increased risk of organ damage caused by inadequate blood flow during episodes of low blood pressure. Therefore, maintaining stable hemodynamics is crucial for hypertensive patients compared with those with normal blood pressure. Additionally, blood pressure may increase excessively in stressful situations, such as intubation [5], pinning [6], or surgical incision. Elevated blood pressure may cause myocardial ischemia and infarction due to increased cardiac workload [7–9]. In particular, a decrease in diastolic BP causes a reduction in cerebral and myocardial perfusion [10]. Moreover, intraoperative hypotension has been reported to be associated with postoperative complications, including acute kidney and myocardial injury [11, 12].

The conventional method for intraoperative total intravenous anesthesia (TIVA) involves the use of propofol and opioids. TIVA provides a feasible setting for intraoperative evoked potential monitoring of brain tumors. Propofol, the first-choice anesthetic drug for the induction and maintenance of anesthesia, has drawbacks, including vasodilation, decreased cardiac output, and a higher likelihood of hypotension in high-risk patients [13, 14].

Remimazolam, a newer benzodiazepine used for the induction and maintenance of general anesthesia [15–17] or procedural sedation [18, 19], acts as a positive allosteric modulator of the γ-aminobutyric acid subtype A (GABA_A_) receptor via the benzodiazepine-binding site [20]. Benzodiazepines have been typically administered to patients with hemodynamic instability or comorbidities to reduce the risk of hypotension during the induction phase [18]. Therefore, it is expected that blood pressure will be maintained more stably when remimazolam is used. However, various studies have reported different results regarding hypotension. We aimed to determine whether propofol or remimazolam leads to stable vital signs during the induction period.

## Methods

### Ethics

This prospective randomized controlled trial was conducted between February 2022 and August 2022. The study protocol (IRB # 4–2021-1456) was approved by the Institutional Review Board of Severance Hospital, Yonsei University Health System (Chairperson Prof. Dr. Jae Hee Cheon, 50–1 Yonsei-ro, Seodaemun-gu, Seoul, Korea; 07/12/2021) and registered with ClinicalTrials.gov (NCT05164146; Principal investigator: Sujung Park, Date of registration: 20/12/ 2021) prior to enrollment. The study was carried out according to the Declaration of Helsinki and Good Clinical Practice guidelines [21]. [21] The patients provided written informed consent on the day before surgery.

### Study population

The inclusion criteria were as follows: age more than 19 years and less than 75 years, history of hypertension, American Society of Anesthesiologists Physical Status (ASA PS) I–III, and a plan for neurosurgery under general anesthesia. The exclusion criteria were as follows: emergency surgery, cardiologic comorbidities other than hypertension, liver failure, or cirrhosis, increased intracranial pressure, mental changes, ambulatory surgery, foreigners, and illiteracy.

### Randomization

A computer-generated randomization table (available at https://www.randomizer.org/form.htm) was used to randomly assign patients to the remimazolam or propofol groups at a 1:1 ratio. Randomization and group assignment were performed by a researcher who did not participate in the data collection.

### Study protocol

All patients received written information about the study on the day before surgery. Upon entering the operating room, the patients were monitored with pulse oximetry, non-invasive arterial blood pressure measurement, electrocardiography, and anesthetic depth measurement (SedLine®; Masimo Corp., Irvine, CA, USA). Furthermore, the systolic pressure, diastolic pressure, mean blood pressure (MBP), and heart rate were recorded at 1 min intervals after the administration of sedative drugs. The patients received 0.1 mg of glycopyrrolate prior to the infusion of remifentanil and remimazolam or propofol using a commercial syringe pump (Agillia; SB Medica SRL, Casalpusterlengo, Italy) [22].

In the propofol group, anesthesia was induced using propofol (target-controlled infusion (TCI), Marsh model) and remifentanil at effect-site concentrations of 4 mcg.ml^−1^ and 4 ng.ml^−1^, respectively. In the remimazolam group, anesthesia was induced using remifentanil at an effect-site concentration of 4 ng.ml^−1^ (TCI, Minto model) and remimazolam at a flow rate of 6 mg kg^−1^.h^−1^, as per the manufacturer’s recommendations. In both groups, sufficient propofol and remimazolam were administered to maintain the depth of electroencephalography-based anesthesia with SedLine^®^ Patient State Index (PSI™) 40 as the target. The opioids were maintained using remifentanil at an effect-site concentration of 4 ng.ml^−1^ (TCI, Minto model). Neuromuscular blockade was induced using intravenous rocuronium (0.6 mg.kg^−1^) after the loss of consciousness. At 3 min after rocuronium administration, endotracheal intubation was attempted using a video laryngoscope and an endotracheal tube in both groups. No other invasive procedures were performed for recording blood pressure, aside from intubation.

Hypotension was defined as a decrease in MBP to < 80% of baseline values (recorded just before anesthetic infusion) in 13 min following the administration of propofol or remimazolam. In cases of MBP < 60 mmHg, ephedrine and norepinephrine were administered as appropriate.

### Study endpoint

The primary outcome measure was the incidence of hypotension. The secondary outcome variables were changes in blood pressure and heart rate during the induction period and changes in PSI™.

### Sample size calculation

According to Liu et al. [23], the incidence of hypotension during induction was 16.7% for remimazolam and 43.3% for propofol. Therefore, the significance level (alpha) was fixed at 0.05 in the formula; when the power (1-β) was 80%, the number of samples considering a dropout rate of 10% was 50 per group, with a total of 100 participants.

### Statistical analysis

Continuous and categorical variables are reported as the mean ± standard deviation and number (percentage), respectively. Continuous variables were analyzed using Student’s t-test or Mann–Whitney U test, as appropriate. Categorical variables were analyzed using chi-squared test or Fisher’s exact test. Hemodynamic variables were assessed utilizing a linear mixed model, with record identification as a random effect and group, time, and interaction between group and time as fixed effects, utilizing an unstructured covariance matrix. All statistical analyses were performed using R package version 4.2.1. (http://www.R-project.org; R Foundation for Statistical Computing, Vienna, Austria). Statistical significance was set at *P* < 0.05.

## Results

**Fig. 1:**
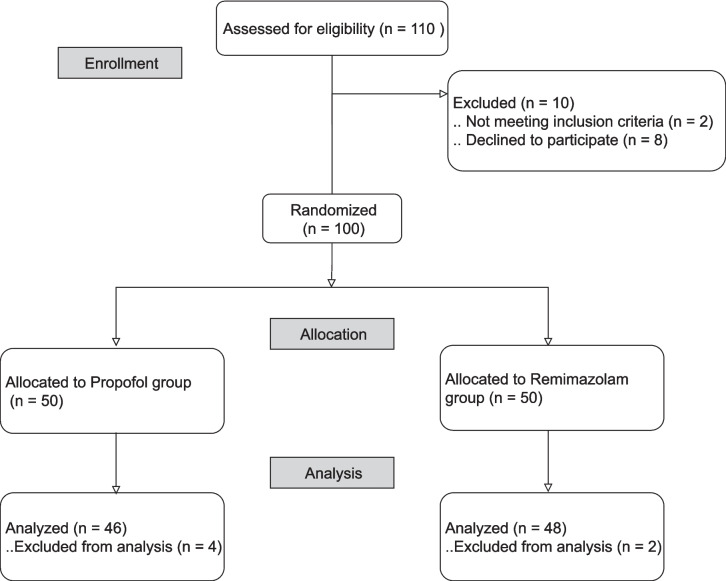
Patient enrollment flowchart. Of 110 patients slated for elective neurosurgery with general anesthesia, 2 patients were disqualified based on the inclusion criteria, and 8 patients opted not to join. Another 6 patients were excluded from the study because of technical issues, resulting in 94 patients for the final analysis

**Fig. 2:**
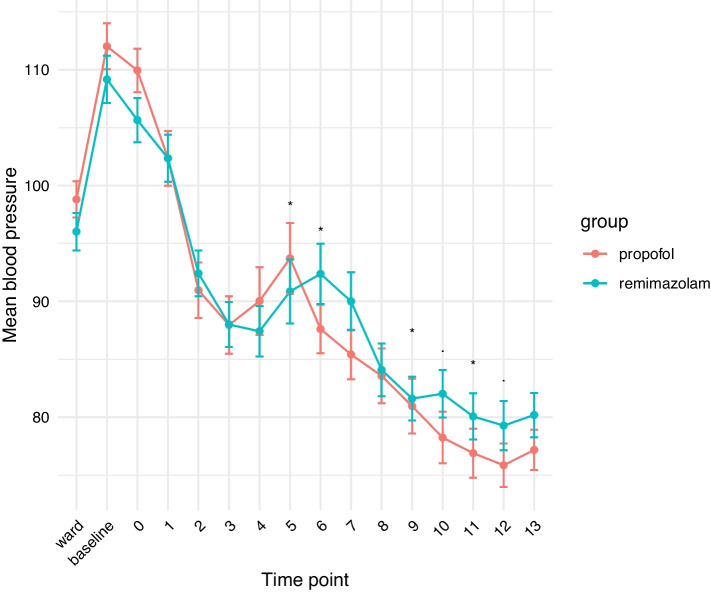
MBP during the induction period. Among 94 patients scheduled for elective neurosurgery under general anesthesia, the MBP was examined in the ward, at baseline (immediately before administering the sedative drug in the operation room), and from 0 min (start of drug administration) to 13 min (13 min after administering the sedative drug). MBP values represent the estimated means from the linear mixed model with standard error. *P < 0.05, • < 0.1 in post-hoc analysis

A total of 100 patients were enrolled; however, 6 patients dropped out due to temporary defects in SedLine®. Only data from 94 patients were included in the final analysis (Fig. 1). There were no significant differences in patient characteristics between the two groups (Table 1). The incidence of hypotension in the propofol group was 91.3%, whereas the remimazolam group exhibited an incidence of 85.4% (*P* = 0.057). Changes in the mean arterial pressure over time showed significant differences between the groups (*P* = 0.029). Post-hoc analysis revealed that the MBP was significantly different between the two groups at 5, 6, 9, and 11 min after the administration of anesthetics (Fig. 2). When examining the minimum MBP of each patient, no significant difference was observed between the two groups. The minimum MBP was 68 (64–78) in the propofol group, whereas it was 72 (64–82) in the remimazolam group (*P* = 0.400).

**Table 1:** Characteristics of patients in the propofol and remimazolam groups

		Propofol	Remimazolam	*P* value
		(*n* = 46)	(*n* = 48)	
Height (cm)		162.2 ± 8.3	162.8 ± 7.6	0.726
Weight (kg)		65.4 ± 11.7	67.37 ± 12.4	0.424
Sex	female	24 (52.2)	26 (54.2)	0.999
	male	22 (47.8)	22 (45.8)	
Age (years)		64.3 ± 7.6	60.9 ± 9.5	0.060
ASA PS	II	19 (41.3)	28 (58.3)	0.149
	III	27 (58.7)	20 (41.7)	
Surgery type - Removal of brain tumor		31 (67.4)	36 (75.0)	0.767
- MVD		4 ( 8.7)	5 (10.4)	
- Brain biopsy		3 ( 6.5)	3 ( 6.3)	
- Etc		8 (17.4)	4 ( 8.3)	
Total dose of sedative drug (mg)		203.89 ± 39.13	30.62 ± 9.20	
Total dose of remifentanil (mcg)		155.91 ± 25.50	158.27 ± 25.75	0.657

Data are presented as the number of patients (percentage) or mean ± standard deviation. Etc (other surgery types) includes procedures such as battery change for deep brain stimulation, ventriculoperitoneal shunting, removal of plate from bone, stereotactic surgery, and encephaloduroarteriosynangiosis

*ASA PS *American Society of Anesthesiologists Physical Status, *MVD *Microvascular decompression

Table 2 shows the subgroup analysis of the primary outcome. We compared the incidence of hypotension among different types of antihypertensive medications. Notably, a significant number of patients were taking several types of antihypertensive medications. Nevertheless, in each comparison, there was no significant difference in the incidence of hypotension. At most time points, the median heart rate was higher in the remimazolam group than in the propofol group (Table 3).

**Table 2:** Incidence of hypotension in patients under anesthesia according to the antihypertensive medication

	Overall	Propofol	Remimazolam	*P* value
ARB	59 (89%)	30 (91%)	29 (88%)	> 0.900
CCB	49 (83%)	23 (88%)	26 (79%)	0.500
ACEi	1 (100%)	0 (0%)	1 (100%)	> 0.900
Beta-blocker	9 (90%)	3 (100%)	6 (86%)	> 0.900
Diuretics	10 (91%)	6 (100%)	4 (80%)	0.500

Data are presented as the number of patients (percentage)

*ARB* Angiotensin receptor blocker, *CCB* Calcium channel blocker, *ACEi* Angiotensin-converting enzyme inhibitor

**Table 3:** Comparison of heart rate changes over time in patients administered propofol or remimazolam

	Propofol	Remimazolam	*P* value
Baseline	68.5 (61.2–77.8)	71.0 (62.0–77.0)	0.500
0 min	69.0 (60.0–78.0)	72.0 (60.0–76.8)	0.700
1 min	65.5 (59.0–74.5)	69.0 (59.8–77.2)	0.300
2 min	62.0 (57.2–68.8)	66.0 (57.8–74.0)	0.110
3 min	60.0 (51.8–68.5)	68.5 (63.0–79.0)	0.001
4 min	66.5 (58.2–79.0)	69.5 (63.0–78.5)	0.300
5 min	72.5 (62.2–81.5)	78.0 (65.0–91.0)	0.089
6 min	70.0 (64.2–81.8)	80.5 (66.8–87.2)	0.044
7 min	67.5 (61.2–76.8)	78.5 (67.8–85.2)	0.008
8 min	68.5 (62.0–77.0)	75.0 (66.0–83.0)	0.068
9 min	68.0 (61.2–75.0)	74.0 (63.0–84.0)	0.049
10 min	65.5 (60.2–74.8)	75.0 (62.0–84.2)	0.008
11 min	65.0 (59.0–76.0)	73.0 (62.8–83.0)	0.021
12 min	66.0 (59.0–75.0)	73.5 (63.0–80.0)	0.038
13 min	64.0 (58.2–75.0)	72.5 (63.8–80.5)	0.025

Data are presented as the median (interquartile range)

**Fig. 3:**
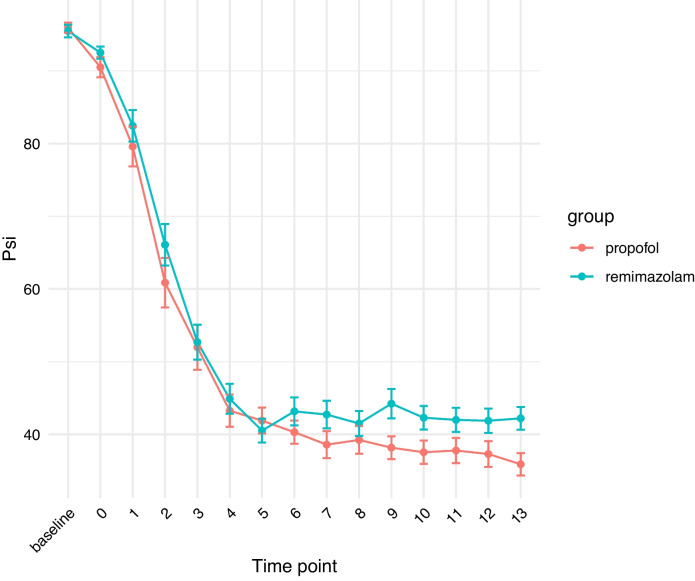
Comparison of PSI™ values at each time point. The PSI™ on the SedLine® monitor was recorded at baseline (just before the administration of the sedative drug in the operating room), and starting at the moment the drug was initiated (0 min) and continuing up to 13 min after administering the drug. The desired PSI™ was achieved 3 min after sedative drug administration and was maintained until the study endpoint at 13 min

When examining the depth of sedation, the target PSI™ was reached at 3 min after the administration of sedative drugs and was maintained until the end of the study at the 13 min mark (Fig. 3). At the 9 and 13 min marks following drug administration, the remimazolam group exhibited significantly high PSI™ values. However, both groups maintained an appropriate depth of anesthesia (PSI™ 25–50), making these differences clinically insignificant.

## Discussion

In this study, we examined the vital signs of patients during the induction period with either propofol or remimazolam. Throughout the observation period, the MBP values of both groups were similar. Notably, at most time points, the median heart rate was significantly higher in the remimazolam group than in the propofol group. This finding would be helpful for selecting an anesthetic for patients with cardiovascular risk factors.

Dai et al. [17] conducted a comparative analysis of the safety and effectiveness of remimazolam versus propofol during anesthetic induction in patients classified as ASA PS I or II. The study demonstrated a lower incidence of hypotension in the remimazolam group than in the propofol group. However, in their study, propofol was administered as a bolus, and the definition of hypotension was different. Choi et al. [24] compared hemodynamic data between remimazolam- and propofol-based TIVA, which, similar to our study, involved administering propofol through TCI and utilizing the manufacturer-recommended dosage of remimazolam. In the study conducted by Choi et al. [24], changes in the MBP before and after induction were not significantly different between the two groups, which are consistent with the results of our study.

Several researchers have reported an increased heart rate after remimazolam administration [24, 25]. However, it remains unclear whether remimazolam increases sympathetic activity or maintains a balance between sympathetic and parasympathetic activities [26]. Therefore, further research on this topic is required. Caution is needed when administering remimazolam to patients with heart conditions who could be endangered by fluctuations in the heart rate.

In a study conducted by Xu et al. [27], the heart rate was higher when sufentanil was used in conjunction with remimazolam instead of propofol. On sedation, it has been reported that remimazolam is less likely to cause bradycardia [28, 29]. In a pilot study conducted with children, it was also shown that remimazolam may contribute to reduced bradycardia [30]. Given these consistent findings, remimazolam may be recommended for patients who are susceptible to bradycardia. Kheterpal et al. [31] demonstrated that patients undergoing chronic angiotensin-converting enzyme inhibitor and angiotensin receptor blocker (ACEi/ARB) treatments with diuretics experienced more episodes of hypotension. An increased incidence of hypotension may be anticipated among ACEi/ARB users; however, this was not the case in the present study. This outcome may be attributed to differences in the number of patients per subgroup and variations in blood pressure management among them.

This study has some limitations. First, we did not monitor vital signs throughout the surgery but examined blood pressure from the start of induction until just before the start of the surgery. However, because hypotension has been most frequently observed during this period [1], examination of blood pressure changes in this period allowed us to understand the effects of the drugs on blood pressure. Additionally, after the start of surgery, changes in blood pressure varied depending on the extent of the surgery, indicating that one must consider the possibility of blood pressure changes due to surgical stimuli rather than the effects of the drugs themselves. Second, although we aimed for a target PSI™ of 40 using the SedLine® monitor, we were unable to achieve this target perfectly. However, the interquartile range (IQR) of the PSI™ observed every minute fell within the manufacturer's recommended range for an appropriate anesthetic depth (25–50), suggesting that an adequate level of anesthetic depth was maintained throughout the observation period. Finally, even in patients taking antihypertensive medications, the extent of preoperative blood pressure control can affect blood pressure changes during surgery. However, this aspect was not investigated, which is a limitation of the study.

## Conclusions

Remimazolam and propofol could result in a similar incidence of hypotension when used for TIVA. Neurosurgery may be performed interchangeably with this approach. Further studies on other types of surgery are warranted to evaluate the effects of remimazolam.

## Data Availability

The datasets analyzed in the current study are available from the corresponding author upon request.

## Abbreviations

MBP: Mean blood pressure
GABA_A_: γ-Aminobutyric acid subtype A
ASA PS: American Society of Anesthesiologists Physical Status
TIVA: Total intravenous anesthesia
PSI™: Patient State Index
TCI: Target-controlled infusion
IQR: Interquartile range
ACEi: Angiotensin-converting enzyme inhibitor
ARB: Angiotensin receptor blocker

## References

[1] Reich DL, Hossain S, Krol M, Baez B, Patel P, Bernstein A (2005). Predictors of hypotension after induction of general anesthesia. Anesth Analg.

[2] Yoon U, Setren A, Chen A, Nguyen T, Torjman M, Kennedy T (2021). Continuation of angiotensin-converting enzyme inhibitors on the day of surgery is not associated with increased risk of hypotension upon induction of general anesthesia in elective noncardiac surgeries. J Cardiothorac Vasc Anesth.

[3] Aslan NA, Vural Ç, Yılmaz AA, Alanoğlu Z (2018). Propofol versus thiopental for rapid-sequence induction in isolated systolic hypertensive patients: a factorial randomized double-blind clinical trial. Turk J Anaesthesiol Reanim.

[4] Malinowska-Zaprzałka M, Wojewódzka M, Dryl D, Grabowska SZ, Chabielska E (2005). Hemodynamic effect of propofol in enalapril-treated hypertensive patients during induction of general anesthesia. Pharmacol Rep.

[5] Oczenski W, Krenn H, Dahaba AA, Binder M, El-Schahawi-Kienzl I, Jellinek H (1999). Hemodynamic and Catecholamine stress responses to insertion of the combitube, laryngeal mask airway or tracheal intubation. Anesth Analg.

[6] Sarna R, Patel S, Singh N, Bloria S, Chauhan R, Meena S (2023). Sterile silicone studs-a nonpharmacologic modality for prevention of hemodynamic response to skull pin insertion: a pilot study. World Neurosurg.

[7] Pedrinelli R, Ballo P, Fiorentini C, Denti S, Galderisi M, Ganau A (2012). Hypertension and acute myocardial infarction. J Cardiovasc Med.

[8] Saheera S, Krishnamurthy P (2020). Cardiovascular changes associated with hypertensive heart disease and aging. Cell Transplant.

[9] Razo C, Welgan CA, Johnson CO, McLaughlin SA, Iannucci V, Rodgers A (2022). Effects of elevated systolic blood pressure on ischemic heart disease: a burden of proof study. Nat Med.

[10] McEvoy JW, Chen Y, Rawlings A, Hoogeveen RC, Ballantyne CM, Blumenthal RS (2016). Diastolic blood pressure, subclinical myocardial damage, and cardiac events. J Am Coll Cardiol.

[11] Walsh M, Devereaux PJ, Garg AX, Kurz A, Turan A, Rodseth RN (2013). Relationship between intraoperative mean arterial pressure and clinical outcomes after noncardiac surgery. Anesthesiology.

[12] Sun LY, Wijeysundera DN, Tait GA, Beattie WS (2015). Association of intraoperative hypotension with acute kidney injury after elective noncardiac surgery. Anesthesiology.

[13] Chang KSK, Davis RF (1993). Propofol produces endothelium-independent vasodilation and may act as a Ca2+ channel blocker. Anesth Analg.

[14] Bang JY, Kim S, Choi BM, Kim TY (2019). Pharmacodynamic analysis of the influence of propofol on left ventricular long-axis systolic performance in cardiac surgical patients. J Korean Med Sci..

[15] Doi M, Morita K, Takeda J, Sakamoto A, Yamakage M, Suzuki T (2020). Efficacy and safety of remimazolam versus propofol for general anesthesia: a multicenter, single-blind, randomized, parallel-group, phase IIb/III trial. J Anesth.

[16] Mao Y, Guo J, Yuan J, Zhao E, Yang J (2022). Quality of recovery after general anesthesia with Remimazolam in patients’ undergoing urologic surgery: a randomized controlled trial comparing Remimazolam with propofol. Drug Des Devel Ther.

[17] Dai G, Pei L, Duan F, Liao M, Zhang Y, Zhu M (2021). Safety and efficacy of remimazolam compared with propofol in induction of general anesthesia. Minerva Anestesiol.

[18] Pastis NJ, Yarmus LB, Schippers F, Ostroff R, Chen A, Akulian J (2019). Safety and efficacy of Remimazolam compared with placebo and Midazolam for moderate sedation during bronchoscopy. Chest.

[19] Tang Y, Yang X, Yu Y, Shu H, Xu J, Li R (2022). Remimazolam versus traditional sedatives for procedural sedation: a systematic review and meta-analysis of efficacy and safety outcomes. Minerva Anestesiol.

[20] Kilpatrick GJ, Mcintyre MS, Cox RF, Stafford JA, Pacofsky GJ, Lovell GG (2007). CNS 7056: a novel ultra-short-acting Benzodiazepine. Anesthesiology.

[21] World Medical Association (2013). World Medical Association declaration of Helsinki: Ethical principles for medical research involving human subjects. JAMA.

[22] Fahy BG, Chau DF (2018). The technology of processed electroencephalogram monitoring devices for assessment of depth of anesthesia. Anesth Analg.

[23] Liu T, Lai T, Chen J, Lu Y, He F, Chen Y (2021). Effect of remimazolam induction on hemodynamics in patients undergoing valve replacement surgery: a randomized, double-blind, controlled trial. Pharmacol Res Perspect.

[24] Choi JY, Lee HS, Kim JY, Han DW, Yang JY, Kim MJ (2022). Comparison of remimazolam-based and propofol-based total intravenous anesthesia on postoperative quality of recovery : a randomized. J Clin Anesth.

[25] Sekiguchi R, Kinoshita M, Kawanishi R, Kakuta N, Sakai Y, Tanaka K (2023). Comparison of hemodynamics during induction of general anesthesia with remimazolam and target-controlled propofol in middle-aged and elderly patients: a single-center, randomized, controlled trial. BMC Anesthesiol.

[26] Hasegawa G, Hirata N, Yoshikawa Y, Yamakage M (2022). Differential effects of remimazolam and propofol on heart rate variability during anesthesia induction. J Anesth.

[27] Xu Q, Wu J, Shan W, Duan G, Lan H (2023). Effects of remimazolam combined with sufentanil on hemodynamics during anesthetic induction in elderly patients with mild hypertension undergoing orthopedic surgery of the lower limbs: a randomized controlled trial. BMC Anesthesiol..

[28] Tang Y, Gao X, Xu J, Ren L, Qi H, Li R (2023). Remimazolam besylate versus propofol for deep sedation in critically ill patients: a randomized pilot study. Crit Care.

[29] Tang Y, Yang X, Yu Y, Shu H, Xu J, Li R (2022). Remimazolam versus traditional sedatives for procedural sedation: a systematic review and meta-analysis of efficacy and safety outcomes. Minerva Anestesiol..

[30] Hirano T, Kimoto Y, Kuratani N, Cavanaugh D, Mason KP (2023). Remimazolam for pediatric procedural sedation: results of an institutional pilot program. J Clin Med.

[31] Kheterpal S, Khodaparast O, Shanks A, O’Reilly M, Tremper KK (2008). Chronic angiotensin-converting enzyme inhibitor or angiotensin receptor blocker therapy combined with diuretic therapy is associated with increased episodes of hypotension in noncardiac surgery. J Cardiothorac Vasc Anesth.

